# Structural Characterization of the *Lactobacillus Plantarum* FlmC Protein Involved in Biofilm Formation

**DOI:** 10.3390/molecules23092252

**Published:** 2018-09-04

**Authors:** Gianluca D’Abrosca, Antonella Paladino, Emilio Cuoco, Rosangela Marasco, Severina Pacifico, Simona Piccolella, Valeria Vastano, Margherita Sacco, Carla Isernia, Lidia Muscariello, Gaetano Malgieri

**Affiliations:** 1 Department of Environmental, Biological and Pharmaceutical Science and Technologies, University of Campania-Luigi Vanvitelli, Via Vivaldi, 43-81100 Caserta, Italy; gianluca.dabrosca@unicampania.it (G.D.A.); paladino.anto@gmail.com (A.P.); emilio.cuoco@unicampania.it (E.C.); rosangela.marasco@unicampania.it (R.M.); severina.pacifico@unicampania.it (S.P.); simona.piccolella@unicampania.it (S.P.); valeria.vastano@unicampania.it (V.V.); margherita.sacco@unicampania.it (M.S.); carla.isernia@unicampania.it (C.I.); 2 Institute of Chemistry of Molecular Recognition, CNR, via M. Bianco, 9-20131 Milan, Italy

**Keywords:** lactic acid bacteria, probiotics, biofilm, LytR-CpsA-psr

## Abstract

*Lactobacillus plantarum* is one of the most predominant species in the human gut microbiota of healthy individuals. We have previously characterized some probiotic features of *L. plantarum* LM3, as the high resistance to different stress, the binding ability toward some extracellular matrix proteins and plasminogen and the immunomodulatory role of the surface expressed adhesin EnoA1. We have also identified the *flmA*, *flmB* and *flmC* genes, coding for putative proteins named FlmA, FlmB and FlmC, whose null mutations partially impaired biofilm development; the *L. plantarum* LM3–6 strain, carrying a deletion in *flmC*, showed a high rate of autolysis, supporting the hypothesis that FlmC might be involved in cell wall integrity. Here, we report the in-silico characterization of ΔTM-FlmC, a portion of the FlmC protein. The protein has been also expressed, purified and characterized by means of CD spectroscopy, ICP-mass and UHPLC-HRMS. The obtained experimental data validated the predicted model unveiling also the presence of a bound lipid molecule and of a Mg(II) ion. Overall, we provide strong evidences that ΔTM-FlmC belongs to the LytR-CpsA-Psr (LCP) family of domains and is involved in cell envelope biogenesis.

## 1. Introduction

Studies on biofilm development are of great interest for the impact they may have on different aspects of the human life [1,2,3,4]. Exploitation of microbial biofilm has been accomplished in different fields, including bioremediation and biotechnological production processes [3,5,6]. Contrariwise, biofilms developed by pathogens represent a serious problem for human health; indeed, cells resident into biofilms show an increased resistance to environmental stress, to immune-response and to antimicrobial molecules [7]. In a continuous struggle to discover new antibiotics or new formulations [8,9,10,11], the scientific community has been making big efforts to search for molecules capable to treat biofilm-depending chronic infections [12,13]. On the other hand, commensals belonging to the microbiota of healthy individuals may grow in the sessile form associated to the mucus layer in the gut and to particulate surfaces in the gut lumen, acting as innate immune protectors of the underlying epithelial cells and as antagonists of pathogens by means of competitive exclusion mechanisms [14,15]. Moreover, *Lactobacillus* biofilms growing on vaginal or intestinal epithelia may have a protecting role against sexually transmitted, urinary or intestinal infections in healthy individuals [15,16,17]. Therefore, one of the features searched in probiotic strains to be selected for treatment of vaginal or intestinal dysbiosis is the ability to form biofilms in the environmental conditions encountered either in the vagina or in the colon [4,18]. Indeed, it is well known that the physico-chemical conditions have significant influences on the ability of the different strains to form biofilms [17].

Probiotics, whose features are defined on the strain basis, mainly belong to the group of the lactic acid bacteria of the genus *Lactobacillus* and *Bifidobacterium*. Among these, some strains of *Lactobacillus plantarum*, one of the most predominant species in the human gut microbiota of healthy individuals, have been defined as good performing probiotic microorganisms [19,20,21,22]. *L. plantarum* is able to colonize a wide range of environmental niches for its high metabolic versatility, contrariwise to the majority of probiotic lactobacilli, which are highly specialized for growth in a limited number of conditions [23]. Comparative genomics of various *L. plantarum* strains revealed high genomic diversity, based on the presence of genomic islands containing mosaic modules of genes for carbohydrate utilization, shedding light on the capability of various *L. plantarum* strains to colonize different environmental niches [24]. Based on these features, *L. plantarum* is largely used as starter in food industry for vegetable, meat, fodder and milk fermentation and for the development of probiotic formulations [24,25].

We have previously characterized important probiotic features of *L. plantarum* LM3, as the high resistance to different stress, assessed in a multiple test for a simulated gastrointestinal transit (Vastano, personal communication), the binding ability toward some extracellular matrix proteins and plasminogen and the immunomodulatory role of a surface expressed adhesin, namely the EnoA1 protein, also involved in biofilm development [26,27,28,29]. Moreover, we have identified the *flmA*, *flmB* and *flmC* genes, coding for putative proteins named FlmA, FlmB and FlmC, whose amino acid sequences show significant percentage of identity with *Streptococcus mutans* BrpA (biofilm regulator protein A) [30,31]. BrpA is located on the cell surface and is involved in maintaining the structure of the cell wall through the regulation of autolysins. More recently BrpA has been indicated as a virulence factor in different pathogenic streptococcal species [32,33]. Indeed, *L. plantarum* strains carrying null mutations in the *flmA*, *flmB* and *flmC* genes, were partially impaired in biofilm development; one of them, the *L. plantarum* LM3–6 strain, carrying a deletion in *flmC*, showed a high rate of autolysis, supporting the hypothesis that FlmC might be involved in cell wall integrity [30].

Interestingly, FlmA, FlmB and FlmC, as well as BrpA, contain a highly conserved domain, that appears to belong to the LytR-CpsA-Psr (LCP) family of domains. The LCP family of cell envelope-associated transcriptional attenuators gained attention upon the discovery that some members of this family influence various virulence factors as well as antibiotic resistance of important human pathogens. Moreover, the LCP family seems to play a role in bacterial cell envelope maintenance [31,34,35,36]. Members of the LCP family were demonstrated to be magnesium-dependent phosphotransferase, responsible of the linkage of the anion cell-wall attached polymers to the peptidoglycan [37]. In this framework, the characterization of FlmC, especially if integrated with studies on its structural features, appears to be of interest, being *L. plantarum* a protective probiotic as quoted above.

In this study, we report the expression, purification and characterization of the *L. plantarum* ΔTM-FlmC, a portion of the FlmC protein. In order to describe the structural characteristics of this protein, we first performed an in-silico analysis and a computational modelling study. The protein has been then characterized by means of CD spectroscopy, ICP-mass and UHPLC-HRMS. The experimental data obtained validated the predicted model unveiling also the presence of a bound lipid molecule and of a Mg(II) ion. Overall, we provide strong evidences that ΔTM-FlmC belongs to the LCP family and is involved in cell envelope biogenesis.

## 2. Results and Discussion

### 2.1. Sequence Analysis and ΔTM-FlmC Expression 

Recently, we have identified the *flmA*, *flmB* and *flmC* genes from *L. plantarum* LM3, coding for putative proteins named FlmA, FlmB and FlmC [30]. The secretome database attributes to these ORF a role of regulatory proteins and a function of cell envelope-related transcriptional attenuator; it is also known that all of them contain a N-terminal trans-membrane anchor domain. Interestingly, the three *L. plantarum* proteins contain in their C-terminal portion highly conserved regions that closely resemble the family of the LytR-CpsA-Psr (LCP) domain. Specifically, among the three Flm proteins, we have chosen to characterize FlmC, as the deletion of the corresponding gene (*flmC*) results in a *L. plantarum* strain (*L. plantarum* LM3–6) with a compromised biofilm development and increased autolytic activity phenotypes; the deletion of the other *flm* genes only hampered biofilm development but not the autolytic activity [30].

**Figure 1:**
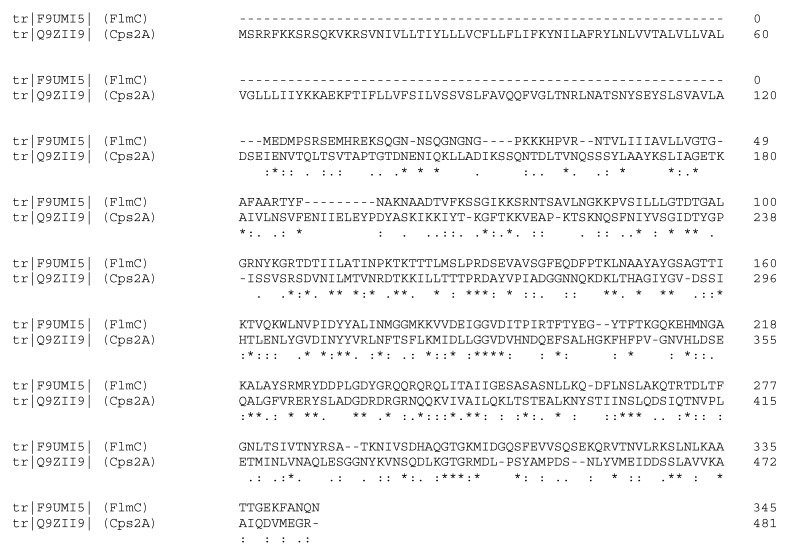
The primary sequence of *L. plantarum* FlmC aligned with the protein Cps2A from *S. pneumoniae*.

Figure 1 proposes the primary sequence of FlmC and its alignment, using the server ClustalO, with the LCP domain of the protein Cps2A. The server aligns the two proteins providing 50% of similarity and 29% of identity between the two sequences, especially in the region starting from residue V81 of FlmC and residue L219 of Cps2A.

This alignment underlines the high similarity of the two proteins in the portion that contains the LCP domain.

The X-ray structure of the protein ΔTM-Cps2A from *Streptococcus pneumonia*, obtained by deletion of the transmembrane portion of Cps2A and containing the LCP domain and an accessory domain, was published in 2011 [37] shedding light on the structural details and function of this domain. 

**Figure 2:**
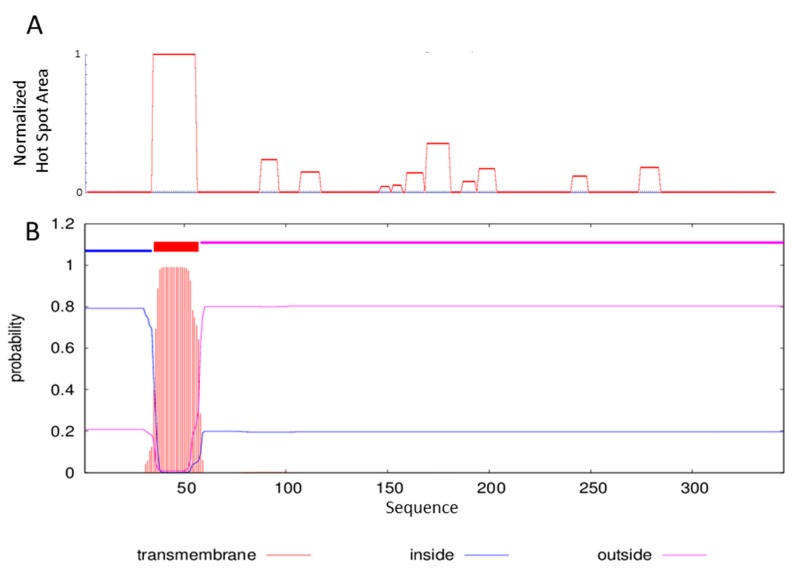
FlmC aggregation profile using the servers AGGRESCAN (**A**). Prediction of transmembrane helices by TMHMM server (**B**).

We thus decided to further characterize the *L. plantarum* FlmC by expressing and purifying a portion of the protein. One of the requisite to attend in selecting the appropriate portion of the protein to express is to avoid inclusion of low-complexity regions or hydrophobic residue rich sequences at the C- or N-termini [38]. For this reason, we have studied the aggregation profile of the selected protein using the servers AGGRESCAN [39] and TMHMM [40], whose results are reported in Figure 2.

The region spanning residues 33–56 is clearly characterized by a highly aggregation prone sequence (Figure 2-panel A). The analysis performed with the server TMHMM capable of predicting the formation of transmembrane helices in proteins outlines for the same region the possibility of a transmembrane domain (Figure 2-panel B).

Therefore, we expressed and purified the sequence spanning residues 81 to 335 obtaining the deletion mutant FlmC_81-335_ (ΔTM-FlmC). For high-level protein production purposes, we expressed the protein in *E. coli* BL21(DE3) that has the advantage of being deficient in both *lon* and *ompT* genes coding for proteases and it is compatible with the T7 *lacO* promoter system. For purification purposes, the protein was produced with a hexahistidine tag that was proven not to consistently impact on the terminal structure of recombinant proteins.

### 2.2. ΔTM-FlmC Structural Model

**Figure 3:**
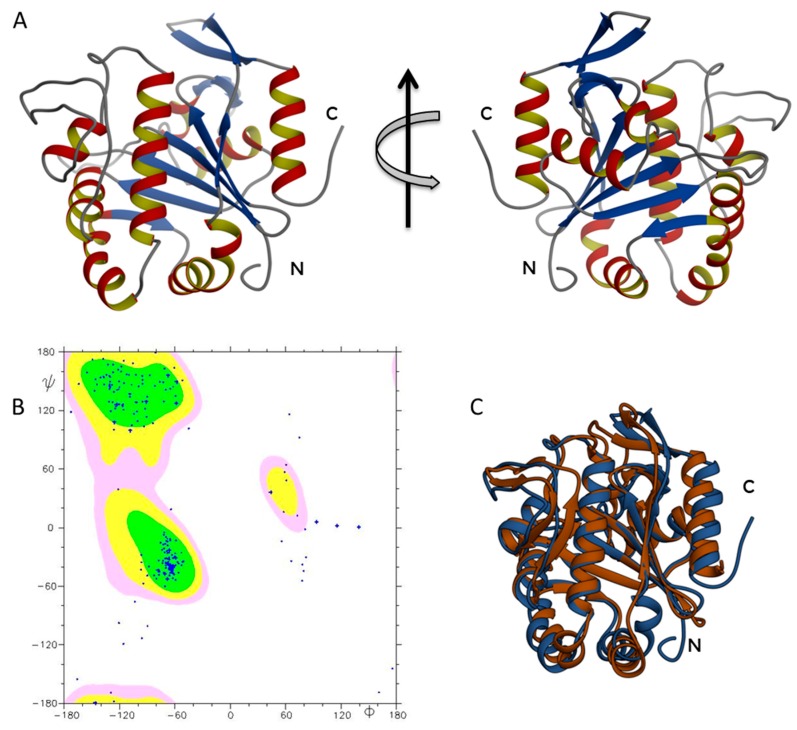
(**A**) ΔTM-FlmC structural model; (**B**) the Ramachandran plot confirms the good quality of ΔTM-FlmC predicted model; (**C**) ΔTM-FlmC superimposition with the crystallographic structure of Cps2A.

The three-dimensional structure represents a very informative and useful tool to understand the functional features of the examined proteins. ΔTM-FlmC three-dimensional structure (Figure 3) was modelled using the I-TASSER algorithm [41] with the primary sequence as input data, using as a template a member of the LCP protein family (3TFL pdb code), taking advantage of a 29% sequence identity for the full-length sequence alignment among the two systems (up to 37% identity at the β-sheet level). In particular, the structure prediction by I-TASSER relies on template known protein structures and the prediction procedure is based on matching the query sequence against a non-redundant sequence database. The computational model of ΔTM-FlmC gave a C-scores of 1.53 indicating a good quality of the predicted model. The c-score that estimates the quality of I-TASSER predicted models typically ranges between −5 and 2, with higher value indicating models with a higher confidence. 

The obtained ΔTM-FlmC structure (Figure 3A) was further analysed by evaluating the Ramachandran plot (Figure 3B), using the software PROCHECK e MOLMOL. Over 97% of the residues were either in favoured or allowed regions denoting the good quality of the predicted model. The ΔTM-FlmC model was then energetically minimized by using Gromacs [42].

In order to gain further insight into the stability of the modelled structure we ran 5 ns MD simulation as reported in the methods section. The last frame of the trajectory was used for further analyses.

As expected, ΔTM-FlmC adopted a compact structure similar to the LCP domain so far characterized (Figure 3C). Overall, ΔTM-FlmC exhibits the typical topology of this domain with most of the hydrophobic amino acid residues buried in the interior and many of the polar residues on the surface. Hydrophobic interactions are a major force that drive protein folding and structure by forcing hydrophobic side chains to closely associate in such a way that they result shielded from polar solvents [43,44,45]. The tertiary structure of ΔTM-FlmC had an architecture with a central sheet composed by six-strands surrounded on both faces by ten (total) -helices held together by short non-helical regions (Figure 3A). 

The overall architecture provided a structural support for a hydrophobic pocket (Figure 3A,B) between the main-sheet and helices 3–7 in which, in the Cps2A protein [37], a polyisoprenoid phosphate lipid is inserted.

**Figure 4:**
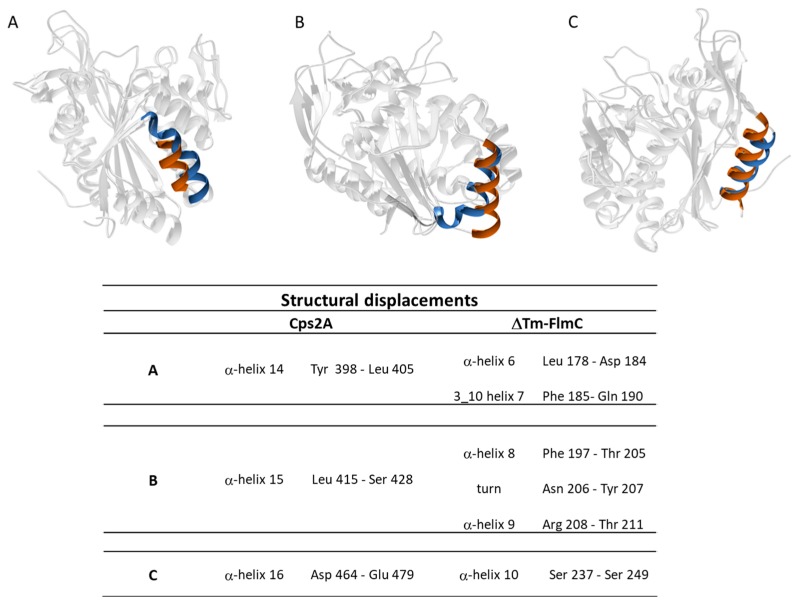
Panel **A**, **B** and **C** show the main structural differences between ΔTM-FlmC (blue) and Cps2A (red); the structural elements shown are reported in the table.

A comparison of ΔTM-FlmC predicted model with the crystallographic structure of Cps2A (PDB code: 2XXP—Figure 3C) indicates that both proteins show a classical globular fold. The back-bone superposition of ΔTM-FlmC model with the portion encompassing residues 219–481 of the crystallographic structure showed an RMSD of 2.1 Å. Predictable minor differences were found between the two protein structures, being local rearrangements the response to preserve the global fold in presence of differences in amino acids composition [46,47]. Therefore, we compared the orientation and length of the secondary structure elements in the modelled structure with those of the reported structure of the LCP domain, respectively. As reported in Figure 4, in ΔTM-FlmC α-helix 6 and 3–10 helix 7 (L178-D182 and F183-Q190, respectively-panel A), α-helix 8 and α-helix 9 (F197-T205 and R208-T211-panel B) and α-helix 10 (S237-S249-panel C) slightly differ in terms of length and axis orientation with respect to the comparable secondary structural elements of Cps2A. Notably, the two proteins share a similar hydrophobic cleft, in terms of structural features. Overall, the hydrophobic pocket in which is located the polyisoprenoid phosphate lipid, considering also the sidechain orientation of the distal and proximal histidine residues (H64, H93), does not show any significant structural difference. 

### 2.3. ΔTM-Flmc Binding Pocket

Predictions of the binding pocket and docking studies were carried out on the final protein conformation of the MD simulation (rmsd = 0.4 nm on all atoms along the 5 ns simulation time). A combinatorial computational strategy was used in order to better validate our modelled system.

First, the identification of pockets on protein surface was performed by LIGSITE [48] and 3 dligandsite [49], independently and prior to docking studies. The two chosen predictors rely on different search algorithms, the former based on solvent accessibility analysis by Connolly surface approach, the latter predicting ligand binding sites based on ligands present in homologous protein structures. The combination of the different outcomes yielded to the definition of a protein cavity running along the protein, involving both charged amino acids from the top of the β-sheet (D29, R145, R147, R157) and hydrophobic residues buried at the α-helix level (namely I165, I168, I169, L187, V204). It is interesting that very bulky and hydrophobic amino acids localized at the inner helical region of the identified pocket result highly aligned/overlapping to residues that make contacts with prenyl moiety onto homologous Cps2A complex.

**Figure 5:**
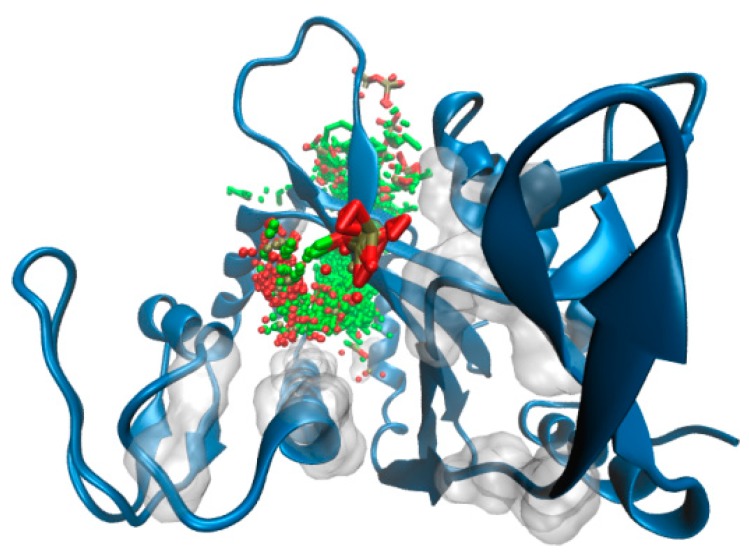
SwissDock binding predictions. The full set of octaprenyl pyrophosphate docking poses are shown in sticks at the binding pocket. Transparent surface is used to pinpoint residues that define the binding cavity. ΔTM-FlmC is rendered in blue cartoons.

**Figure 6:**
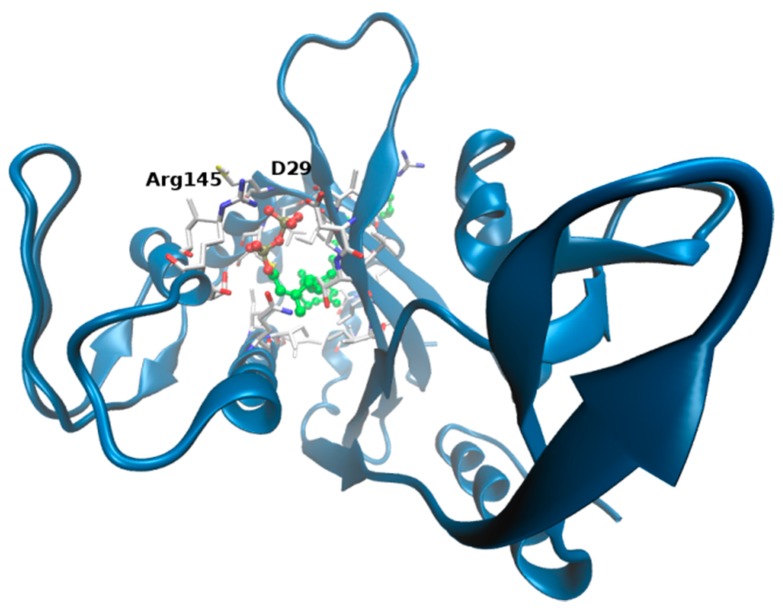
Docking models. Best ranked ligand pose predicted by SwissDock: ΔTM-FlmC is shown in blue cartoons, octaprenyl pyrophosphate in CPK and amino acids within 3 Å from the ligand in white sticks. Interacting residues at the top of the cavity are labelled (D29, R145). See main text.

To further support these findings (and provide a visual idea to the model), we investigated putative binding poses by means of docking studies (Figure 5). Octaprenyl pyrophosphate ligand (from 3TFL PDB) was docked onto the protein, with no constraints on the binding pocket using SwissDock [50]. More than 250 binding modes of the ligand into ΔTM-FlmC cavity were generated. From Figure 5 and Figure 6, it is evident that phosphate heads in all poses mostly overlap at the top of the β-sheet interacting with D29, R145, in a similar rearrangement to what was observed for Cps2A homologous protein.

Presumably, on the other side, long aliphatic chain inserts into the channel delimited by the β-sheet and the long α-helix, making contacts with I165, L187 localized at the very buried cavity. The best ranked binding pose predicted is displayed in Figure 6 (the docking pose is associated to a free energy of binding of −12.7 kcal/mol).

An additional validation of the bound complex was also taken from GalaxyWEB [51]. This server is able to provide predictions of ligands that are likely to bind to the protein and their relative binding poses. Figure S1 shows binding site interactions analysis predicted by Galaxy on the first docking pose. It is interesting to underline that the main interactions made by the phosphate moieties at the top of the binding site are conserved between the two complexes.

### 2.4. Circular Dichroism Characterization and Validation of the Obtained Model

**Figure 7:**
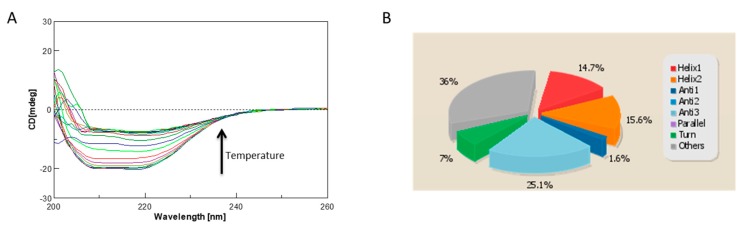
(**A**) CD spectra of ΔTM-FlmC reported as function of temperature; (**B**) Secondary structure content evaluated using the CD data by the server BeStSel.

One strategy for assessing accuracy is to cross-validate the calculated structures or models using properties not considered in the computation. We performed a cross-validation analysis for ΔTM-FlmC predicted models using the Circular Dichroism (CD) to further validate the predicted model. CD is an excellent tool for rapid determination of secondary structure and folding properties of synthesized peptides [8,11,52,53] and of proteins [54,55] that have been obtained using recombinant techniques or purified from tissues. The most widely used applications of protein CD are to determine whether an expressed, purified protein is folded, or if a mutation affects its conformation or stability [47]. In addition, it can be used to study protein and peptide in teractions [56]. The CD spectrum of ΔTM-FlmC (Figure 7A) is characteristic of a well-structured protein containing both -helical and -sheet secondary structure. We estimated using the CD data the protein secondary structure content by using the server BeStSel (Figure 7B). The data indicate that the secondary structure content of our purified protein well fits the content of secondary structure evaluated for the I-TASSER calculated model. In fact, purified ΔTM-FlmC-helix content calculated from the CD spectra is 30.3% while the structures content is 26.7%. These values are in a good agreement with the -helix and -structures amounts obtained evaluating the predicted model using the software MOLMOL and DSSP.

Overall, our analysis demonstrated that the I-TASSER computationally obtained model represents a realistic picture of the tertiary structure that the purified ΔTM-FlmC protein adopts in solution. The stability of ΔTM-FlmC has been also evaluated by monitoring the CD signal at 222 nm (Figure 7A). ΔTM-FlmC irreversible thermal unfolding encompassing the temperature range between 278 and 353 K gave a midpoint temperature of unfolding at around 315 K confirming an overall structural stability of the purified protein.

### 2.5. ΔTM-FlmC Domain Binds a Lipid Molecule and a Magnesium Ion

Kaway et al. [37] showed that the Cps2A protein co-purifies with a polyisoprenoid phosphate lipid located in a hydrophobic pocket between the main -sheet and -helices 3–7. They hypothesized that their protein had bound the lipid when heterologously expressed in *E. coli*, confirming its affinity for a lipid-linked capsule precursor in *S. pneumoniae*. The lipid was built as a monotrans, octa-cis decaprenyl-phosphate. For the ΔTM-FlmC protein, the server I-TASSER, able to foretell binding sites in the calculated models, predicted an extensive hydrophobic binding pocket that could easily accommodate an analogous lipid molecule.

Figure 6 shows ΔTM-FlmC cavity: it is composed by hydrophobic side chains (Figure S1) of residues that, while not completely identical to those of Cps2A, are conserved across the LCP family and are likely to give to the pocket the same hydrophobic character. Moreover, ΔTM-FlmC cavity appears to have a comparable diameter so that a lipid molecule can be easily accommodated in it with only few interactions with the protein (Figure 6). Figure S1 shows the protein residues that constitute the hydrophobic pocket and are likely to be involved in the interaction with the lipid molecule.

As for Cps2A, also in the case of ΔTM-FlmC charged residues (R50, R145 and R157) surround the area where is located the phosphate head group of the prenyl, stabilizing the binding.

Given these evidences, we have investigated whether also ΔTM-FlmC co-purified with a similar lipid. We performed an organic solvent extraction as in Harrison et al. [57] and evaluated the products via TLC (thin-layer chromatography) analysis that suggested the presence of a lipid (data not shown). The total ion current (TIC) chromatogram (Figure S2A), acquired in positive ion mode in the range *m*/*z* 100–1500, showed a protonated molecular ion at *m*/*z* 775.5789. Based on previous findings [1], the occurrence of a decaprenyl phosphate (dpr-P) ligand tightly bound to the protein was hypothesized. Indeed, the [M + H]^+^ ion was in accordance with an in-source formed dehydro dpr-P (Figure S2A), whose proposed structure was as depicted. The detection of the doubly charged ion at *m*/*z* 387.2863 ([M−2H]^2−^), in accordance with the molecular formula C50H81O4P^2−^, recorded in the TOF-MS spectrum acquired in negative ion mode, likely confirmed dpr-P presence (Figure S2B). 

Members of the LCP family were proved to bind a magnesium ion which appears to be necessary for their magnesium-dependent phosphotransferase activity. 

As proposed by Kawai and co-workers [37] a Mg(II) ion binds the Cps2A protein with an octahedral geometry. The protein interacts with the metal ion using the two aspartate residues D234 and D246. The magnesium ion plays a crucial role in the function of the protein as its loss, as well as the mutation of one of the coordinating aspartates, leads to a reduction in the phosphatase activity. ΔTM-FlmC three-dimensional structural model evidences how the two homologous positions are occupied also in this protein by two aspartates (D16 and D29), suggesting for these residues the same role in coordinating a magnesium ion. We have thus explored the presence of metal ions bound to ΔTM-FlmC by ICP-MS that revealed how also this protein binds a Mg(II) ion.

Our experiments conducted on different dilutions of the protein solution indicated the presence of magnesium ions whose concentrations varied as function of the protein concentrations (Figure S3), thus reinforcing the idea that D16 and D29 are likely to be involved in the coordination of a Mg(II) ion.

## 3. Materials and Methods

### 3.1. Bioinformatics 

All the used amino acid sequences were retrieved and analysed using the BLAST software (http:// blast.ncbi.nlm.nih.gov/Blast.cgi). Alignments were performed by Clustal Omega at EMBnet-CH (http://www.ebi.ac.uk/Tools/msa/clustalo). FlmC aggregation profile was evaluated using the server AGGRESCAN and transmembrane helix content evaluated by the server TMHMM [40]. ΔTM-FlmC three-dimensional structure has been modelled by I-Tasser [41]. Molecular dynamics simulations were run using Gromacs package (v.4.5.5) [58]. The secondary structure content from the CD data were evaluated using BestSel [59]. The structure has been validated using Procheck [60] and visualized using Pymol [61] and Chimera [62].

### 3.2. Expression and Purification of ΔTM-FlmC from L. plantarum

*L. plantarum* LM3 chromosomal DNA was used as template in a PCR reaction and a fragment of the *flmC* gene from nucleotides 241 to 1006 was amplified. Due to the presence of the *Nde*I restriction site in the *flmC* gene, a two steps-cloning strategy was used. The MLF1for/MLF2 rev and MLF3 for/MLF4 rev pairs of primers were used in the first and in the second step, respectively. The two DNA fragments of 402 and 363 bp, coding for aa residues V_81_–M_215_ and M_215_–A_335_, respectively, were amplified by PCR and cloned in pET22b(+) (Novagen). The 402 bp fragment was cloned in *Nde*I/*Not*I sites, yielding the pFlmC_134_ plasmid. The 363 bp fragment was cloned in the *Nde*I site of the recombinant pFlmC_134_, yielding the pFlmC_254_ plasmid, which was selected in *E. coli* TOP10. After sequencing, the recombinant pFlmC_254_ plasmid was used to transform *E. coli* BL21 (DE3) for FlmC_81–335_ (hereafter ΔTM-FlmC) expression. High protein yield was obtained by growing cells in 100 mL of liquid LB at 37 °C up to OD_600_ = 0.2. Cells were then centrifuged at 4.000× g for ten minutes and the pellet was re-suspended in 1L M9 medium and grown at 37 °C with shaking up to OD_600_ = 0.6, before being induced with 1 mM IPTG and incubated at 16 °C for 2 h. Cells were harvested by centrifugation at 4000× *g* for 30 min and washed with 50 mL of 20 mM Tris-HCl, pH 8.0, before a final centrifugation step at 4000× *g* for ten minutes. The cell pellet was then re-suspended in 50 mL buffer A (50 mM Tris-HCl, pH 8.0, 200 mM NaCl, 20 mM imidazole) before sonication for 5 min on ice by a 15 s on, 15 s off cycle. The lysed cells were clarified by centrifugation at 20,000× *g* for 30 min. The cell free extract was loaded onto a 3 mL Ni-charged resin (Biorad), for His-tagged affinity chromatography. The protein was eluted with 10 mL of buffer B (50 mM Tris-HCl, pH 8.0, 200 mM NaCl, 500 mM imidazole). The presence of ΔTM-FlmC was monitored by absorbance at 280 nm and confirmed by analysis of apparent molecular weight of the eluted protein by SDS-PAGE. UV-vis spectroscopy measurements were conducted using a UV-1700 Spectrometer (Shimadzu, Japan) [9] with 1 cm matched quartz cuvettes at room temperature in the wavelength range 200–500 nm.

### 3.3. Circular Dichroism

Circular Dichroism (CD) analysis was performed using a JASCO-815 CD (Jasco, USA) spectropolarimeter equipped with Peltier temperature control. ΔTM-FlmC samples were prepared in 20 mM Tris, 200 mM NaCl, pH 8.0. The data were collected using a quartz cuvette with a 1 cm path-length in the 190–260 nm wavelength range, a bandwidth of 1 nm, scanning speed of 50 nm min^−1^ and normalized against reference spectra to remove the buffer background contribution. The CD spectra have been de–convoluted by using the server BeStSel [59].

Thermal unfolding was followed recording CD spectra measured at 5 K intervals in the 278–353 K range. After the final measurement at 353 K, the sample was cooled back to 298 K and a final spectrum recorded.

### 3.4. 3D Structural Models

The 3D models for ΔTM-FlmC were obtained on the basis of its amino acid sequence using the I-TASSER software. I-TASSER (Interactive Threading ASSEmbly Refinement) is a computational method that combines all three conventional methods for structure modelling: comparative modelling, threading and ab initio modelling [41]. The c-score is a confidence value used to evaluate the quality of I-TASSER predicted models. It is estimated on the basis of the significance of threading template alignments and on the convergence parameters of the structure assembly simulations. The c-score ranges typically from −5 to 2, with higher scores indicating models with higher confidence. The obtained models were evaluated and visualized using the software PROCHECK [60], PyMol [61] MOLMOL [63] and Chimera [62]. Secondary structure estimation using the predicted models was performed using the software DSSP [64]. 

### 3.5. Molecular Dynamics

In order to garner insights into the stability of the modelled structure we run molecular dynamics simulation using Gromacs package (v.4.5.5) [42] with the Amber99 force field [65]. The ΔTM-FlmC protein model was centred in triclinic boxes allowing a 1 nm distance from each box edge and solvated by explicit solvent (TIP3P model), ending with 10493 water molecules. Counterions were randomly added to neutralize the system. First, the system was energy minimized using the steepest descent approach, followed by an equilibration phase where water molecules and protein heavy atoms were position restrained. The unrestrained systems were kept in an NPT ensemble, at constant temperature of 300 K by the velocity rescaling thermostat and at a pressure of 1 bar by the Berendsen thermostat. Electrostatic interactions were evaluated using the particle mesh Ewald method [66] and Lennard-Jones forces by a cut-off radius of 0.9 nm. Bond lengths involving hydrogen were restrained by the LINCS algorithm [67]. The time step was set to 2 fs and periodic boundary conditions were applied in all three dimensions. Protein structure simulation were run with no Mg (II) ion added.

Production run was carried for 5 ns of simulation time and the last frame of the trajectory has been used for the successive structural analyses.

Specifically, predictions of the binding pocket and docking studies were carried out on the final protein conformation of the MD simulation (rmsd = 0.4 nm on all atoms along the 5 ns simulation time).

### 3.6. ICP-MS

Mg(II) ion concentrations were measured via ICP-MS Agilent 7500ce (Agilent Technologies, Inc., USA), equipped with ORS tech interference/reaction cell to reduce polyatomic interferences. Instrument performances were checked using proper Tuning Solution (AGILENT^®^ until the setting related to sensitivity and interference parameters were optimized. Interferences were tested through Interference Check Solutions (AGILENT^®^) in order to check the efficient functioning of ORS system.

Instrumental drift was monitored in continuum using Y-Tb internal standard with constant concentration. The analytical precision and accuracy for repeated quantifications of sample solution, international and internal standards (Agilent solutions EPA 200.8 Validated Standards, Agilent Technologies, Inc., USA), were better than 10%. Detected concentrations exceeded by at least one order of magnitude the limits of detection (LOD) and quantification (LOQ), according to Long and Winefordner [68]. Experiments were conducted in triplicate and for each sample different dilutions were analysed.

### 3.7. Lipid Extraction 

The recombinant ΔTM-FlmC solution (20 mM sodium phosphate and 0.2 M NaCl at pH 6.8) was acidified with HCl in order to completely unfold the protein. The solution was then filtered using Amicon filters (Merck KGaA, Darmstadt, Germany) and the flow through was mixed with CH_3_Cl and CH_3_OH to obtain a final ratio of 2:2:1. The sample was incubated under stirring for 2 h. After centrifugation at 3750× *g* for 10 min, the lower organic fraction was recovered and dried. The resulting extract has been then analysed via TLC using an aluminium sheet Silica gel 60 F_254_ (Merck KGaA, Darmstadt, Germany) developed in a solution of CHCl_3_-CH_3_OH-H_2_O (13:7:2 ratio), sprayed with a solution of H_2_SO_4_-CH_3_COOH-H_2_O (1:20:4 ratio) and charred to visualize the organic compound.

### 3.8. UHPLC-HRMS Analyses

A Shimadzu NEXERA UHPLC system (Shimadzu, Japan) was used with a Luna^®^ Omega C18 column (1.6 μm particle size, 50 × 2.1 mm i.d., Phenomenex, Torrance, CA, USA).

Separation was achieved with a linear gradient of water (A) and acetonitrile (B), both with 0.1% formic acid. Gradient conditions were as follows: 0–5 min, linear from 5% to 55% B; 5–10 min, linear from 55% to 75% B; 10–11 min, linear from 75% to 95% B; 11–13 min, isocratic 95% B. Then, the starting conditions were restored and the column was allowed to re-equilibrate for 1 min. The total run time was 14 min, with a flow rate of 0.5 mL min^−1^ and an injection volume of 2.0 μL. 

MS analysis was performed using a hybrid Q*q*TOF MS instrument, the AB SCIEX TripleTOF^®^ 4600 (AB Sciex, Concord, ON, Canada), equipped with a DuoSprayTM ion source (consisting of both electrospray ionization (ESI) and atmospheric pressure chemical ionization (APCI) probes), which was operated in positive and negative ionization modes. The instrument was controlled by Analyst^®^ TF 1.7 software, while data processing was carried out using PeakView^®^ software version 2.2.

## 4. Conclusions 

The inspiring hypothesis of this study proposes the FlmA, FlmB and FlmC proteins from *L. plantarum* as belonging to the LytR-CpsA-Psr (LCP) protein family, a class of proteins widely distributed among Gram positive bacteria and involved in cell envelop biogenesis and homeostasis [37,69]. Members of the LCP protein family were initially indicated as transcriptional attenuators, due to the pleiotropic effects of null mutations in the corresponding genes but structural and biochemical analyses of LCP proteins from different Gram-positive species support the hypothesis of their role as transporters of anionic polymers, including wall teichoic acids, to the cell wall peptidoglycan [37,70]. 

The analysis of the Flm proteins indicated a high amino acid sequence identity with Cps2A and BrpA (Biofilm regulatory protein A) of *Streptococcus mutans*, both members of the LCP protein family [31,35,36]. In particular, in this study, among the three proteins we chose to characterize FlmC, due to the interesting compromised biofilm development and increased autolytic activity phenotypes, shown by the *L. plantarum* LM3-6 (Δ*flmC*) strain. Mutant strains carrying deletions in the other *flm* genes were only hampered in biofilm development, retaining a wild type autolytic activity [30]. These phenotypes were also found in *S. mutants* strains, carrying single null mutations in *brpA* or *psr* genes, while the deficiency in both genes resulted to be lethal in the conditions used for mutant selection [36]. 

Here we report the characterization of the FlmC protein that reveals the presence at the N-terminus of a hydrophobic fragment, likely a trans-membrane domain and at the C-terminus of a hydrophilic domain that we identify as belonging to the characterizing domain of the LytR-CpsA-Psr protein family. To validate our hypothesis, we have identified the portion of the *L. plantarum* FlmC protein to express, namely ΔTM-FlmC, for structural characterization. ΔTM-FlmC structural model has been then compared to the X-ray structure of the Cps2A protein obtained by Kaway and co-workers [37], evidencing how, apart from minor structural rearrangements, the two proteins appear highly similar. In particular, the two proteins share a hydrophobic cavity that in Cps2A has been proved to host a lipid molecule. Besides that, Cps2A, thanks to two crucial aspartate residues, binds octahedrally to a Mg(II) ion fundamental for its phosphatase activity [37]; the same residues are conserved in ΔTM-FlmC and show in the model a position compatible with the coordination of Mg(II). Accordingly, our experimental data have proved the co–purification with ΔTM-FlmC of a lipid molecule and the presence of a Mg(II) ion. 

The cell wall is of great interest for its role in the communication with the environment, in the defence from a plethora of insults, in the localization of cell-surface expressed virulence factors and in biofilm development. Studies on Lactobacilli biofilms will help to shed light on their involvement in prevention of diseases related to disbiosis conditions, in particular in vaginal or intestinal environments [4,15,16,18]. Our results strongly suggest that the *L. plantarum* FlmC protein is a phosphotransferase, just as the well characterized Cps2A, supporting previous results demonstrating the role of this protein in biofilm development and autolysis activity. 

## Supplementary Materials

The following are available online, Figure S1: Binding site interactions analysys, Figure S2: UHPLC-HRMS analysis, Figure S3: ICP-MS analysis.
