# Supplementary material for: Structural Characterization of the Lactobacillus Plantarum FlmC Protein Involved in Biofilm Formation
Source: Molecules. 2018 Sep 4;23(9):2252. doi: 10.3390/molecules23092252 (PMC6225345; doi:10.3390/molecules23092252)
Supplement: Supplementary file 1 [file molecules-23-02252-s001.pdf]

Structural characterization of the *Lactobacillus plantarum* FlmC protein involved in biofilm formation

Gianluca D'Abrosca[1], Antonella Paladino[1,2], Emilio Cuoco[1], Rosangela Marasco[1], Severina Pacifico[1], Simona Piccolella[1], Valeria Vastano[1], Margherita Sacco[1], Carla Isernia[1], Lidia Muscariello[1*] and Gaetano Malgieri[1*]

[1]Department of Environmental, Biological and Pharmaceutical Science and Technologies, University of Campania – Luigi Vanvitelli, Via Vivaldi, 43 - 81100 Caserta (Italy)

[2]Institute of Chemistry of Molecular Recognition, CNR, via M. Bianco, 9 20131, Milan (Italy).

*****correspondence: Lidia Muscariello (*lidia.muscariello@unicampania.it*) and Gaetano Malgieri *(gaetano.malgieri@unicampania.it)*.

Supplementary Informations

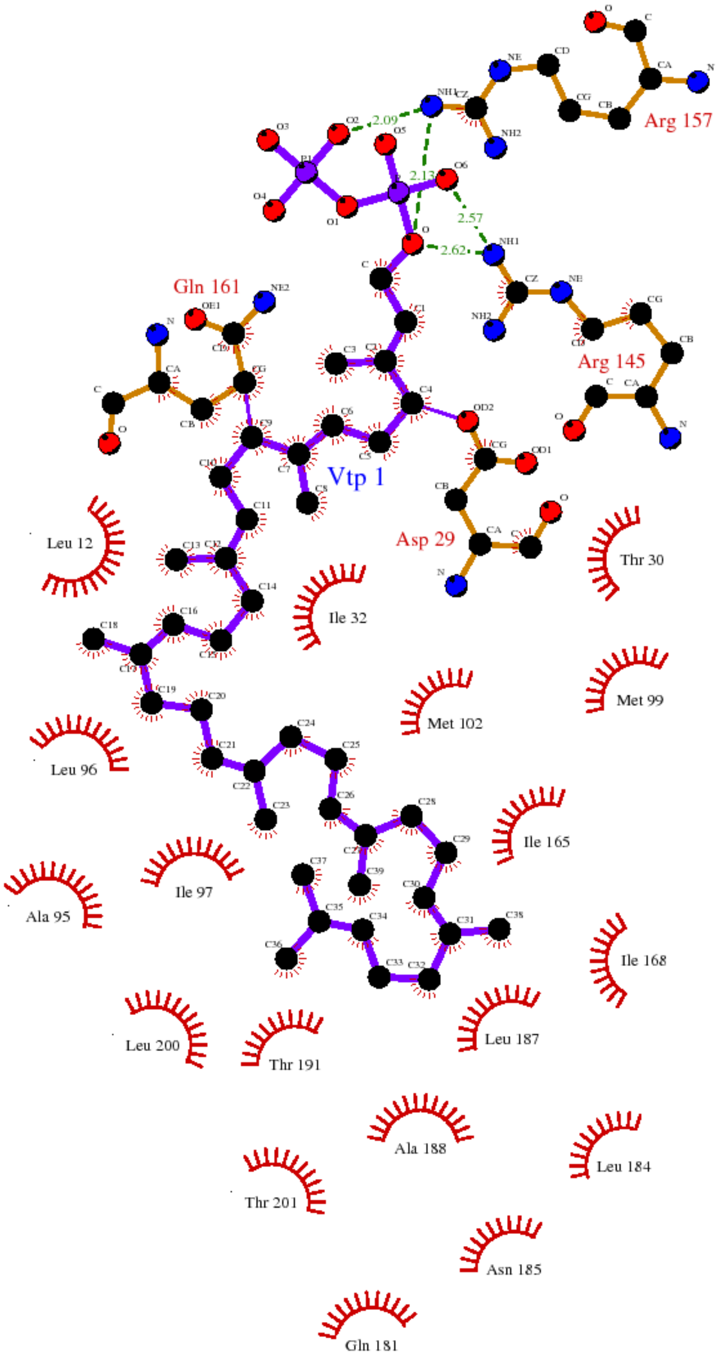

Figure S.I. 1. Binding site interactions analysis predicted by Galaxy on the first docking pose, built using PDB 4DE9 as template for protein-ligand complex.

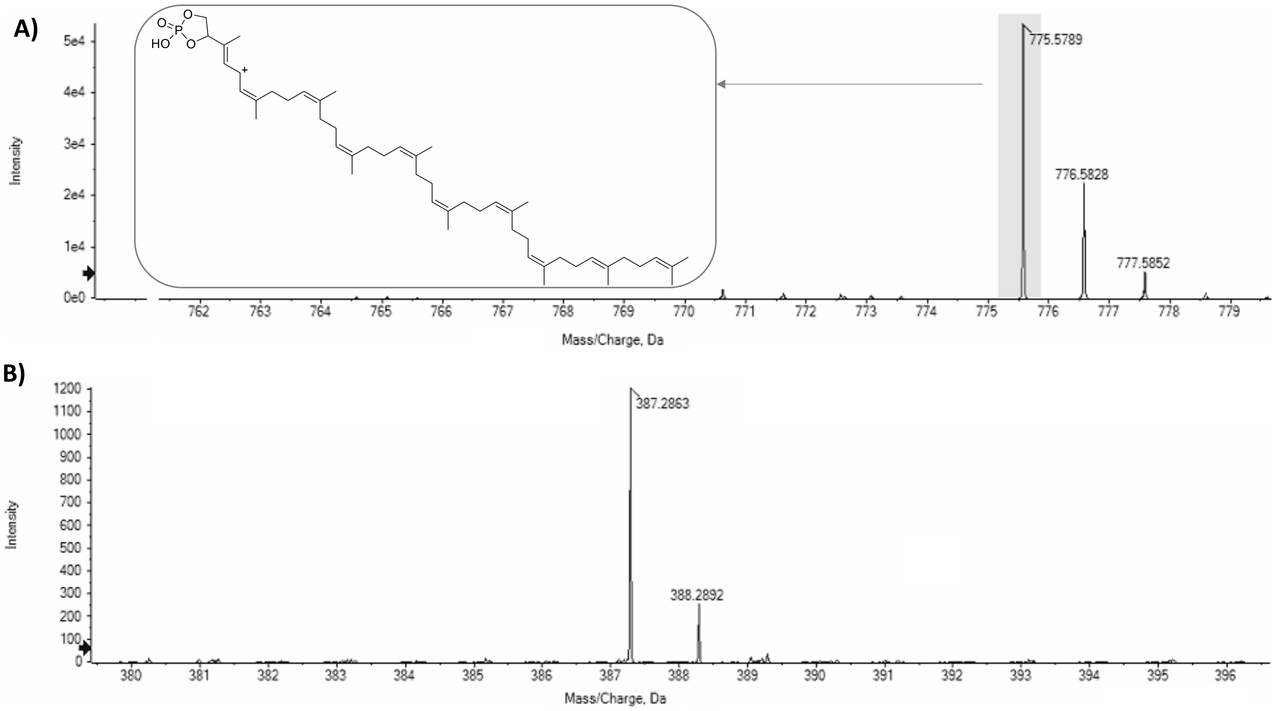

Figure S.I.2 A) TOF-MS acquired in positive ion mode and structure proposed for the ion at m/z 775.5789; B) Doubly charged ion at m/z 387.2863 recorded in negative ion mode acquisition.

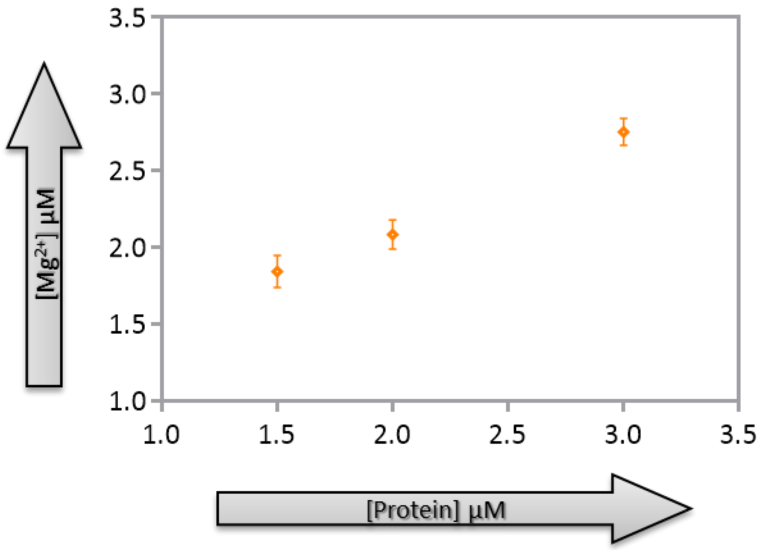

Figure S.I. 3: ICP-MS analysis. Magnesium presence as a function of protein concentration.
